# Multi-Repeated Projection Lithography for High-Precision Linear Scale Based on Average Homogenization Effect

**DOI:** 10.3390/s16040538

**Published:** 2016-04-14

**Authors:** Dongxu Ren, Huiying Zhao, Chupeng Zhang, Daocheng Yuan, Jianpu Xi, Xueliang Zhu, Xinxing Ban, Longchao Dong, Yawen Gu, Chunye Jiang

**Affiliations:** State Key Laboratory for Manufacturing Systems Engineering, Xi’an Jiaotong University, Xi’an 710049, China; zhaohuiying@mail.xjtu.edu.cn (H.Z.); zcp1988123@126.com (C.Z.); 18608160633@wo.cn (D.Y.); 13810533839@163.com (J.X.); xueliang.zhu@foxmail.com (X.Z.); banxinxing405@163.com (X.B.); dlc521@163.com (L.D.); guyawen@stu.xjtu.edu.cn (Y.G.); jiangchunye1992@stu.xjtu.edu.cn (C.J.)

**Keywords:** projection lithography, linear scale, linear displacement sensor, multi-repeated method, average homogenization effect

## Abstract

A multi-repeated photolithography method for manufacturing an incremental linear scale using projection lithography is presented. The method is based on the average homogenization effect that periodically superposes the light intensity of different locations of pitches in the mask to make a consistent energy distribution at a specific wavelength, from which the accuracy of a linear scale can be improved precisely using the average pitch with different step distances. The method’s theoretical error is within 0.01 µm for a periodic mask with a 2-µm sine-wave error. The intensity error models in the focal plane include the rectangular grating error on the mask, static positioning error, and lithography lens focal plane alignment error, which affect pitch uniformity less than in the common linear scale projection lithography splicing process. It was analyzed and confirmed that increasing the repeat exposure number of a single stripe could improve accuracy, as could adjusting the exposure spacing to achieve a set proportion of black and white stripes. According to the experimental results, the effectiveness of the multi-repeated photolithography method is confirmed to easily realize a pitch accuracy of 43 nm in any 10 locations of 1 m, and the whole length accuracy of the linear scale is less than 1 µm/m.

## 1. Introduction

Linear scale is an indispensable component of linear displacement sensors and is defined as the grating structure that is printed on a metal or glass substrate material by photolithography or mechanical scribing. It is the key technology for precision displacement measurements, which are widely used in ultra-precision machining equipment, ultra-precision measuring instruments, and semiconductor manufacturing equipment, where the demand for position accuracy is always increasing. To improve the accuracy of the linear scale, several processing methods have evolved, such as mechanical ruling [1], ion-beam etching [2], nanoimprinting lithography, and UV optical lithography. However, mechanical ruling and ion-beam etching primarily produce plane diffraction gratings, which require deep grooves with strict shapes that can produce non-ideal curved gratings [3]. The roller subdivision and 2PI errors offer challenges to nanoimprinting lithography for high-precision grating; a special rotating exposure to roller mold fabrication was analyzed to fabricate gratings in a large area [4]. Projection lithography is a UV optical lithography technique that presents a solution to the issues associated with precision and resolution; this paper will review a method for manufacturing a high-precision linear scale.

The performance of a projection lithography system is characterized by three major parameters: resolution, depth of focus, and overlay accuracy [5]. When the lithography system configuration is invariable, the quality of the grating lines will only be restricted by focusing accuracy and photoresist resolution, and the inaccuracy of the grating lines include alignment errors, rotational and skew errors of the mask, and mask feature placement errors.

In linear scale projection lithography, in addition to the above problems, the main factors that affect the accuracy of lithography include the static and dynamic positioning accuracy, the parallelism of the focal plane and photoresist plane on the scale, and the uniformity of light intensity distribution. Several methods have adopted the principle of splicing together, in which the patterns are adjacent and printed accurately on the wafer at an accurate distance, such as the step-and-repeat method [6], single-step method [7], and step-and-scan method [8,9], where the *x*- and *y*-direction positioning accuracies are not high. The hexagonal seamless scanning exposure method behaves the advantage for fabrication of large area grating than stitching exposure method in steppers [10], adjacent hexagonal scans overlap partially and integrated doses from successive scans produce uniform exposure over the whole panel, however, we adopt adjacent quadrangular scans overlap by integer times of pitch theoretically, the pitch errors are homogenized by multi-repeated exposure of different position grating on the mask. The influence of lithography processes and methods on the accuracy and errors in the linear scale projection lithography has been rarely reported.

To resolve these issues, a multi-repeated photolithography method with an average homogenization effect that can improve the accuracy of the linear scales is proposed in this paper. The light intensity error function of the focal plane and the accuracy expression of the linear scale are given to describe the operating principles and theory of the proposed method. The influences of internal pitch errors in the mask on the lithography accuracy are simulated and analyzed. It is verified that there is a proportional relationship between the grating strip repetitions and lithography accuracy. The integer times pitch error of the exposure spacing distance is achieved that can adjust the proportion of black and white stripes. The defocus errors caused by a skew focus plane or tilt photoresist plane on the scale are reduced and homogenized.

## 2. Photolithography Principle

**Figure 1:**
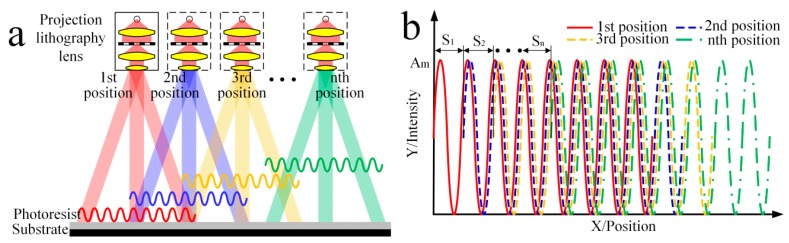
Scheme of the proposed multi-repeated projection lithography, including (**a**) conceptual drawing of the n time exposure; and (**b**) the light intensity distribution of the different exposure distances.

The fundamental principles of multi-repeated projection lithography for a high-precision linear scale are illustrated in Figure 1. The focal plane is calibrated by the lithography lens alignment system to ensure a high-precision grating pattern in the photoresist plane. Divergent light of a certain wavelength from the pulsed xenon lamp passes through the concave lens into a parallel light, which irradiates the grating pattern on the mask to make it accurately projected on the photoresist plane by the lithography lens.

When the irradiation time reaches a set value t at a certain position, a complete exposure process is completed. Meanwhile, the electrical control system triggers the pulsed xenon lamp power off, and the motion control system drives the grating substrate to a distance ($S_{1}$, $S_{2}$, …, $S_{n}$) relative to the lithography lens in one direction. Following the periodic operation process, light intensity eventually is superimposed at different positions to achieve error averaging.

## 3. Theory

### 3.1. Fundamental Theory

Because of the lithography lens numerical aperture, the lithography lens distortion, the optical vignetting effect, the pitch error on the mask and alignment accuracy error will cause projection light intensity pattern errors on the photoresist plane. The light intensity at different positions can be expressed as:

(1) $$I\left(x\right)=\{\begin{array}{c} I_{1}\left(x\right),x\in \left[0,L_{1}\right]\;\\ I_{2}\left(x\right),x\in \left[S_{1},L_{2}+S_{1}\right]\;\\ I_{3}\left(x\right),x\in \left[S_{1}+S_{2},L_{1}+S_{1}+S_{2}\right]\;\\ \vdots \;\\ I_{n}\left(x\right),x\in \left[\overset{n-1}{\underset{i=1}{\sum }}S_{i},L_{n}+\overset{n-1}{\underset{i=1}{\sum }}S_{i}\right] \end{array}$$

where $L_{n}$ is the nth projection length on the photoresist plane (when the projection lens reduction ratio is 0.25, the mask length is 4L), $I_{n}\left(x\right)$ is the nth projection light intensity, $S_{i}$ is the spacing distance between the ith position and the (*i* − 1)th position, and $S_{i}=mp+\Delta_{i}$, m is the integer pitch number of the exposure spacing distance, p is the ideal pitch of the linear scale, and $\Delta_{i}$ is the ith spacing distance error. To better explain the exposure dose distribution of the multi-repeated projection lithography, assume there are km  =$\;n^{\prime }$ pitches on the mask and $I_{ij}\left(x\right)$ is the jth period light intensity of the ith position exposure, *k* is the repeat exposure number of each pitch, and *kmp* = 4*L*. The exposure dose is defined as the light intensity multiplied by the exposure time [11]. The sum expression of the exposure dose under these conditions can be expressed as:

Part B:

(2) $$\{\begin{array}{c} D_{m_{0}}\left(x\right)=\overset{1}{\underset{i=1}{\sum }}I_{ij}\left(x+\Delta_{i}\right)t\\ D_{2m_{0}}\left(x\right)=\overset{2}{\underset{i=1}{\sum }}I_{ij}\left(x+\Delta_{i}\right)t\\ \vdots \;\\ D_{km_{0}}\left(x\right)=\overset{k}{\underset{i=1}{\sum }}I_{ij}\left(x+\Delta_{i}\right)t \end{array}$$

subject to $j=\left(k-1\right)m+m_{0}-\left(i-1\right)m,m_{0}\in \left[1,m\right]$.

Part C:

(3) $$\{\begin{array}{c} D_{km+m_{0}}\left(x\right)=\overset{k+1}{\underset{i=2}{\sum }}I_{ij}\left(x+\Delta_{i}\right)t\;\\ D_{\left(k+1\right)m+m_{0}}\left(x\right)=\overset{k+2}{\underset{i=3}{\sum }}I_{ij}\left(x+\Delta_{i}\right)t\;\\ \vdots \;\\ D_{n-\left(k-2\right)+m_{0}}\left(x\right)=\overset{n-\left(k-2\right)m+k-1}{\underset{i=n-\left(k-2\right)m}{\sum }}I_{ij}\left(x+\Delta_{i}\right)t \end{array}$$

subject to $j=\left(k-1\right)m+m_{0}-\left(i-\left(n-\left(k-2\right)m\right)\right)m,m_{0}\in \left[1,m\right]$.

Part D:

(4) $$\{\begin{array}{c} D_{n-\left(k-1\right)m+m_{0}}\left(x\right)=\overset{n}{\underset{i=n-k+1}{\sum }}I_{ij}\left(x+\Delta_{i}\right)t\\ D_{n-\left(k-2\right)m+m_{0}}\left(x\right)=\overset{n}{\underset{i=n-k+2}{\sum }}I_{ij}\left(x+\Delta_{i}\right)t\\ \vdots \;\\ D_{n-m+m_{0}}\left(x\right)=\overset{n}{\underset{i=n-1}{\sum }}I_{ij}\left(x+\Delta_{i}\right)t \end{array}$$

subject to $j=\left(k-1\right)m+m_{0}-\left(i-(n-1\right))m,m_{0}\in \left[1,m\right]$.

**Figure 2:**
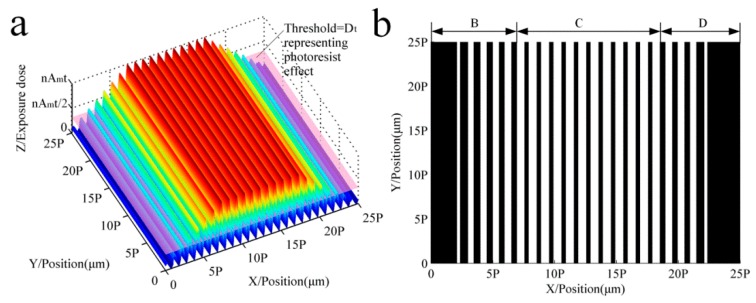
Imaging process using a photoresist. (**a**) The sum of exposure dose under the condition ($S_{1}\;$= $S_{2}\;$= $S_{n}\;$= P); (**b**) The scale grating formed by the developing threshold.

As shown in Figure 2a, there is a critical point in the process of exposure and development technology where the exposure energy is higher than the threshold dose, after which the resist and chromium coating will be washed away to form the black and white stripes, *i.e.*, when [12]:

(5) $$D_{n}\left(x\right)>D_{t}$$

The threshold critical plane is the energy line that intersects with the D(x) function surfaces to form cross-points $x_{i}$, and corresponding to the difference between two adjacent x coordinates is the width of the black and white grating strips; the formula can be expressed as:

(6) $$p_{i}^{\prime }=\Delta x_{i}|X^{-1}\left[D_{i}\left(x\right)=D_{t}\right]$$

where *x* is the function root value of $D_{i}\left(x\right)=D_{t}$ and $p_{i}^{'}$ is the difference between the function root value and also is the pitch formed by the multi-repeated lithography.

From Equations (2)–(4), and Figure 2, it can be seen that the energy amplitude distribution of Part B and Part D is incremented or decremented as the process ends; only the energy curve distribution of Part C is suitable for accuracy error analysis. Then, the theoretical error of this point can be written as:

(7) $$\begin{array}{c} \Delta p_{i}^{\prime }=p_{i}^{\prime }-p\\ =\Delta x_{i}|X^{-1}\left[D_{i}\left(x\right)=D_{t}\right]-p\\ =\Delta x_{i}|X^{-1}\left[\overset{n-\left(k-2\right)m+k-1}{\underset{i=n-\left(k-2\right)m}{\sum }}I_{ij}\left(x+\Delta_{i}\right)t=D_{t}\right]-p \end{array}$$

where $\Delta p_{i}^{'}$ is the error between the ideal pitch and the pitch formed by the multi-repeated projection lithography. Obviously, the average value of the n different exposure doses $D_{n}\left(x\right)$ is smaller than the maximum $Max[D_{n}\left(x\right)]$:

(8) $$\overset{n-\left(k-2\right)m+k-1}{\underset{i=n-\left(k-2\right)m}{\sum }}I_{ij}\left(x+\Delta_{i}\right)t<Max\left[kI_{ij}\left(x+\Delta_{i}\right)t\right]$$

Then, the accuracy comparison of two different lithography methods is given by:

(9) $$\Delta p_{i}^{\prime }<Max\left[\Delta p_{i}\right]$$

subject to i∈[1,n].

Equations (8) and (9) show that the average errors are accumulated and homogenized regularly from the different position and energies in the multi-repeated projection lithography, which are smaller than the maximum errors with respect to the same position and energy in the common projection lithography. Thus, the grating lithography accuracy will be improved.

### 3.2. Mask Error Model

The mask pattern for the linear scale lithography is uniform rectangular stripes. Letting N be the largest diffraction order that passes through the lens, and using the dense space diffraction pattern and Euler’s theorem, the light intensity image is [13]:

(10) $$I_{i}\left(x\right)={\left|a_{0}+2\overset{N}{\underset{j=1}{\sum }}a_{j}cos\left(2\pi jx/p\right)\right|}^{2}$$

where p is the pitch, $a_{0}$ is the DC component, and j is the diffraction order. For the case of equal lines and spaces, the diffraction order amplitudes become:

(11) $$a_{j}=\frac{sin\left(j\pi w/p\right)}{j\pi }$$

In the precise or ultra-precise manufacturing process for the grating mask, micron- or nanometer-level errors are inevitable among different pitches, which does not meet the even spacing requirements of multi-slit Fraunhofer diffraction. According to the analysis of the Fourier optical transfer function, the image light intensity is equivalent to the sum of different frequency harmonics; then, the projection image intensity of separate pitches can be approximated as a single-slit Fourier harmonic expression, and finally, the multi-repeated lithography error can be written as:

(12) $$\begin{array}{c} \Delta p_{i}^{\prime }=\Delta x_{i}|X^{-1}\left[\overset{k+i-1}{\underset{m=i}{\sum }}I_{m}\left(x,p_{i}\right)t=D_{t}\right]-p\\ =\Delta x_{i}|X^{-1}\left[\overset{k+i-1}{\underset{m=i}{\sum }}{\left|a_{0}+2\overset{N}{\underset{j=1}{\sum }}a_{j}cos\left(2\pi jx/p_{i}\right)\right|}^{2}t=D_{t}\right]-p \end{array}\;$$

### 3.3. Exposure Repeated Number Model

Because the resist corrosion threshold to energy required is constant, when the exposure time t is set to a constant value, the exposure number is inversely proportional to light intensity; then, the relationship can be expressed as:

(13) $$D_{constant}\left(x\right)=kI^{k^{\prime }}\left(x\right)t$$

subject to $k\in \left[1,n^{\prime }\right]$, where $I^{k^{\prime }}\left(x\right)$ is the light intensity, corresponding to the repeated number *k*. The maximum average energy of the different repeated exposure numbers can be expressed as:

(14) $$\begin{array}{c} Max\left[\frac{\sum_{m=i}^{k+i-1}I_{m}^{k^{\prime }}\left(x\right)t}{k}\right]<Max\left[\frac{\sum_{m=i}^{k+i-2}I_{m}^{{\left(k-1\right)}^{\prime }}\left(x\right)t}{k-1}\right]<\cdots <Max\left[\frac{\sum_{m=i}^{i+1}I_{m}^{2^{\prime }}\left(x\right)t}{2}\right]<\sum_{m=i}^{i}I_{m}^{\prime }\left(x\right)t \end{array}$$

subject to $i,k\in \left[1,n^{\prime }\right]$.

The average error has the same law, so the maximum average energy error of the different repeated exposed number can be written as:

(15) $$Max\left[\Delta p_{i}^{\prime }\left(RN=k\right)\right]<Max\left[\Delta p_{i}^{\prime }\left(RN=k-1\right)\right]<\cdots <Max\left[\Delta p_{i}^{\prime }\left(RN=2\right)\right]<Max\left[\Delta p_{i}^{\prime }\left(RN=1\right)\right]$$

subject to $i\in \left[1,n^{\prime }\right]$, where RN represents the repeated number. It reflects the influence of repeated exposure number on the lithography accuracy.

### 3.4. Spacing Distance Model

The exposure interval distance is considered as an integer multiple of a pitch in the proposed projection lithography method, which does not accurately reflect the photolithography process. In the actual lithography process, the existence of positioning errors and the demand for error compensation will produce submicron and nanometer error for the exposure interval distance, which need to be discussed because they have a great influence on the lithography accuracy. Thus, we intend to describe the sum expression of exposure dose with non-integral pitch errors:

(16) $$\{\begin{array}{c} D_{1}\left(x\right)=\overset{k}{\underset{m=1}{\sum }}I_{m}\left(x\pm \Delta p\right)t\\ D_{2}\left(x\right)=\overset{k+1}{\underset{m=2}{\sum }}I_{m}\left(x\pm \Delta p\right)t\\ \vdots \;\\ D_{n-k+1}\left(x\right)=\overset{n}{\underset{m=n-k+1}{\sum }}I_{m}\left(x\pm \Delta p\right)t\\ D_{n-k+2}\left(x\right)=\overset{n}{\underset{m=n-k+2}{\sum }}I_{m}\left(x\pm \Delta p\right)t+\overset{1}{\underset{m=1}{\sum }}I_{m}\left(x\pm \Delta p\right)t\\ \vdots \;\\ D_{n-1}\left(x\right)=\overset{n}{\underset{m=n-1}{\sum }}I_{m}\left(x\pm \Delta p\right)t+\overset{k-2}{\underset{m=1}{\sum }}I_{m}\left(x\pm \Delta p\right)t\\ D_{n}\left(x\right)=\overset{n}{\underset{m=n}{\sum }}I_{m}\left(x\pm \Delta p\right)t+\overset{k-1}{\underset{m=1}{\sum }}I_{m}\left(x\pm \Delta p\right)t \end{array}$$

where +Δp and −Δp represent positive deviation and negative deviation of spacing distance, respectively.

## 4. Simulation

The average homogenization effect of the proposed method has proven theoretically that several micro/nano errors have little effect on the projection lithography accuracy; the simulations are carried out as follows.

### 4.1. Influence of Mask Errors

To study the impact of pitch error on the accuracy of multi-repeated lithography, three types of errors are introduced into the simulation: (1) the sum of sinusoid pitch error on the mask is equal to zero; (2) the sum of increasing pitch error on the mask is more than zero; and (3) the sum of decreasing pitch error on the mask is less than zero.

**Figure 3:**
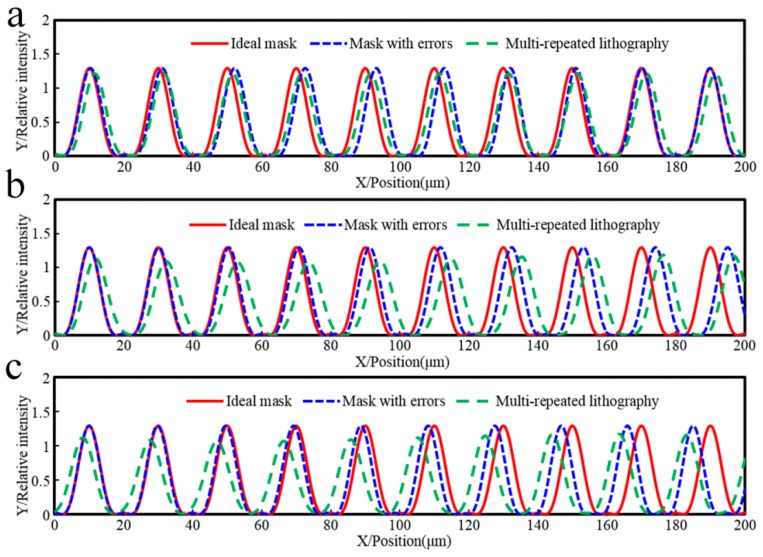
Relative light intensity relationship between a mask with errors, an ideal mask without errors, and the multi-repeated projection lithography method. (**a**) Sinusoid pitch error on mask; (**b**) Increasing pitch error on mask; (**c**) Decreasing pitch error on mask.

**Figure 4:**
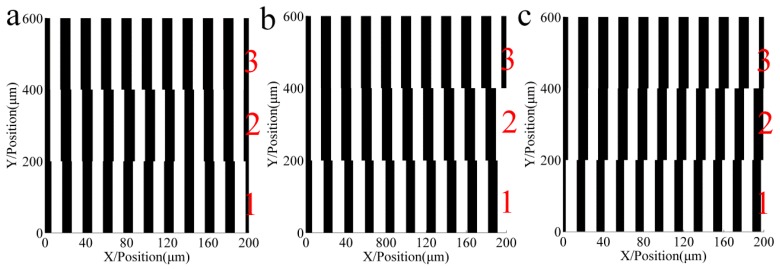
Developing results of the different function errors. Zones 1, 2 and 3 are formed from the intensity of multi-repeated lithography, mask with errors, and ideal mask respectively in (a–c) of Figure 3. (**a**) Grating of sinusoid pitch error; (**b**) Grating of increasing pitch error; (**c**) Grating of decreasing pitch error.

Figure 3 shows the relative light intensity relationship between a mask with errors, an ideal mask without errors, and the multi-repeated projection lithography method. The maximum intensity amplitude of the multi-repeated projection lithography method is smaller than the other two. Assuming the parameter of threshold intensity is 0.25, the linear grating can be formed as in Figure 4, which shows the developing results of the different function errors.

**Figure 5:**
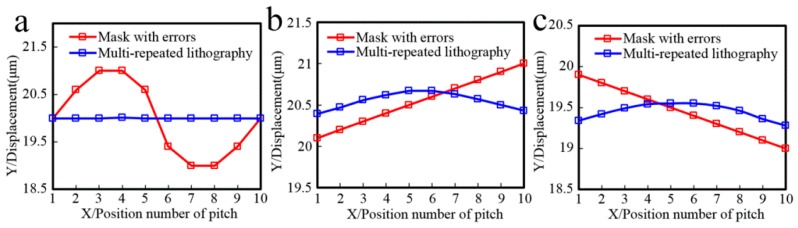
Pitch error comparison of a mask with errors in projection lithography and multi-repeated lithography. The projection lithography pattern pitch error of the mask with error is set respectively as (**a**) $p_{i}\left(x\right)=Asin\left(\omega x\right)+p$; (**b**) $p_{i}\left(x\right)=Kx+p$; and (**c**) $p_{i}\left(x\right)=-Kx+p$.

Figure 5 shows the pitch error comparison of a mask with errors in projection lithography and multi-repeated lithography. When 10 pitch errors of the red thread were sinusoidally distributed at the maximum peak-valley value of 2 µm, the maximum error of the blue thread would be 0.01 µm with the multi-repeated lithography method, and linear scale lithography precision was increased by 99.5%. Meanwhile, by comparing the influence of the proposed method on the pitch type error, while the pitch error of the red thread was set to a linear increasing function distribution or linear decreasing function distribution at the maximum error of 0.9 µm, the maximum error of the blue thread was reduced to 0.28 µm and 0.27 µm, respectively, by the proposed lithography method, and the linear scale lithography precision was increased by 68.9% and 70%, respectively.

Referring to Figure 3 and Figure 5, in comparison with the phase of the mask error projection lithography intensity, when the curvature of pitch error curve was positive in Figure 5, the phase of the multi-repeated lithography intensity was offset to the left in Figure 3; on the other hand, when the curvature of the pitch error curve was negative in Figure 5, the phase of the multi-repeated lithography intensity was offset to the right in Figure 3.

It can be seen from these simulations that the proposed projection lithography process can reduce the influence of mask pitch error on the accuracy of linear scale lithography.

### 4.2. Influence of Repeated Exposure Number

**Figure 6:**
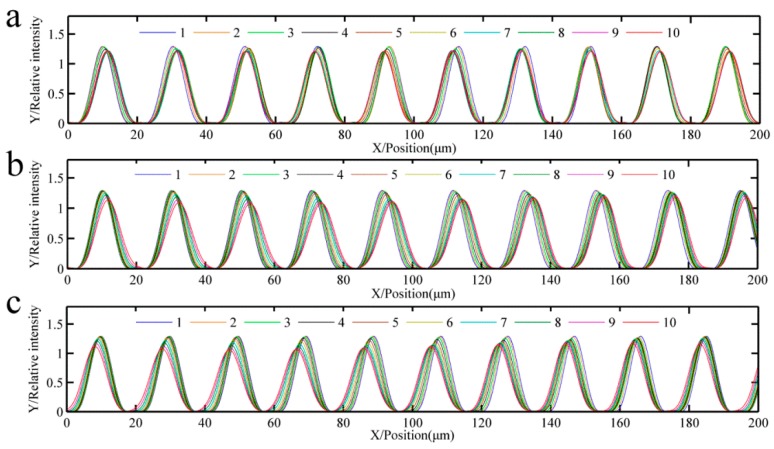
Relative light intensity comparison of 10 different repeat grating; the numbers represent the different repetitions of the proposed method. (**a**) Relative intensity of sinusoid pitch error; (**b**) Relative intensity of increasing pitch error; (**c**) Relative intensity of decreasing pitch error.

**Figure 7:**
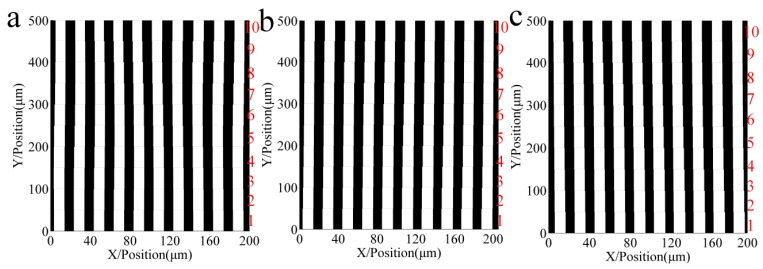
Developing results of the different function errors and the repeat number. Zone 1 to 10 are formed from the respective intensities of the different repetitions of the proposed method. (**a**) Grating of sinusoid pitch error; (**b**) Grating of increasing pitch error; (**c**) Grating of decreasing pitch error.

**Figure 8:**
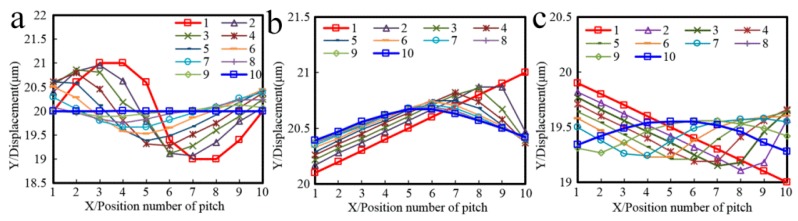
Pitch error comparison of multi-repeated lithography with the different repeat grating numbers. The projection lithography pattern pitch errors of the mask with error are set respectively as (**a**) $p_{i}\left(x\right)=Asin\left(\omega x\right)+p$; (**b**) $p_{i}\left(x\right)=Kx+p$; and (**c**) $p_{i}\left(x\right)=-Kx+p$.

To analyze the influence of repeated exposure number with different grating positions on the accuracy of multi-repeated projection lithography, 10 error repetitions of three types of error functions were applied to the simulation using the light intensity, grating strips, and pitch errors. The three graphs (a–c) of Figure 6, Figure 7 and Figure 8 correspond to each other.

Figure 6 shows the relative light intensity comparison of 10 different repeat gratings. With an increase in the number of repetitions, when the pitch error is incrementally changed, the light intensity phase shifts to the right; in contrast, when the pitch error is decreasing, the light intensity phase shifts to the left. The maximum amplitude of the light intensity decreased. Figure 7 shows the developing results of the different function errors and the repeat number; Figure 7a–c are a sinusoidal function error, linear increasing function error, and linear decreasing function error, respectively. Zone 1 to zone 10 are formed from the intensity of the different repetitions of the proposed method.

Figure 8 shows that the maximum pitch error is reduced with an increase in the repeated exposure number. Figure 8a shows that the maximum sinusoidal error decreased from 2 µm to 0.01 µm, Figure 8b shows that the maximum linear increasing error decreased from 0.9 µm to 0.28 µm, and Figure 8c shows that the maximum linear decreasing error decreased from 0.9 µm to 0.27 µm.

It can be seen from these simulations that increasing the exposure repetitions of the grating strips will reduce pitch errors, which can promote the improvement of the accuracy of the proposed projection lithography process.

### 4.3. Influence of Spacing Distance Errors

To accurately analyze the influence of the spacing distance of non-integral pitch errors on lithography, the ideal intensity period was set to 20 µm, and the spacing distances were set to ±0.1 µm, ±0.2 µm, and ±0.3 µm symmetrically.

**Figure 9:**
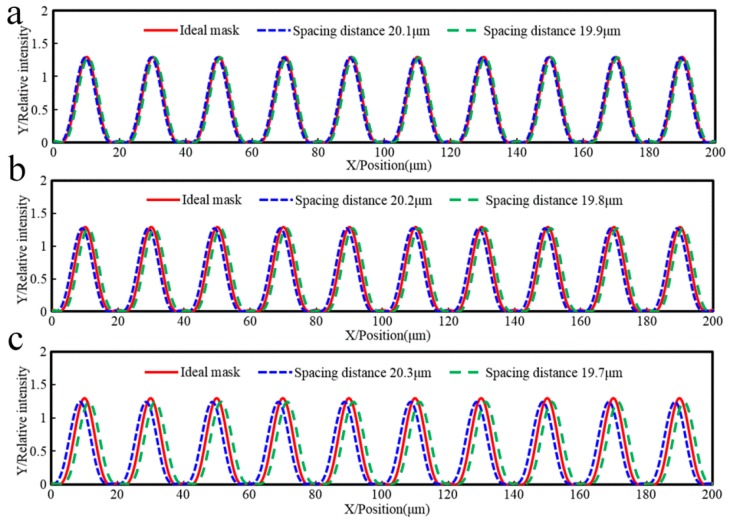
Relative light intensity comparison of the different spacing distance errors: (**a**) spacing distance error is ±0.1 µm; (**b**) spacing distance error is ±0.2 µm; (**c**) spacing distance error is ±0.3 µm.

**Figure 10:**
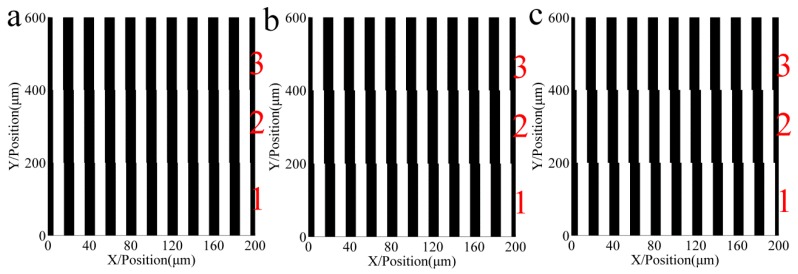
Developing results correspond with spacing distance errors of (**a**) ±0.1 µm; (**b**) ±0.2 µm; and (**c**) ±0.3 µm. Zones 1, 2, and 3 are formed from the intensity of a spacing distance with negative deviation, ideal mask, and spacing distance with positive deviation, respectively.

Figure 9 shows the relative light intensity comparison of the different spacing distance errors. The phase of the positive and negative equivalence error is symmetrically distributed relative to the ideal intensity period. Assuming the parameter of threshold intensity is 0.25, the linear grating can be formed as in Figure 10, which shows that the developing results correspond with spacing distance errors.

It can be seen from these simulations that when the threshold of exposure dose is constant, spacing distance errors and the ratio of black stripes and white stripes are inversely proportional, and the pitch has not been changed in the Table 1, which will affect the accuracy of the half cycle signal. It is necessary to adjust the threshold of exposure dose; then, the ratio of black stripes and white stripes can reach the set value.

### 4.4. Influence of Focal Plane Alignment Errors

To analyze the influence of focal plane alignment errors on the proposed projection lithography accuracy, different alignment accuracies with slope errors were introduced into the simulation. The alignment error of the focal plane in projection lithography includes the slope error and focus depth error. The slope error reflects the parallelism between the focal plane and movement plane of the grating, and the focus depth error reflects the relative position and overlap of the focal plane and grating photoresist plane; the two errors play an important role in the alignment accuracy.

**Figure 11:**
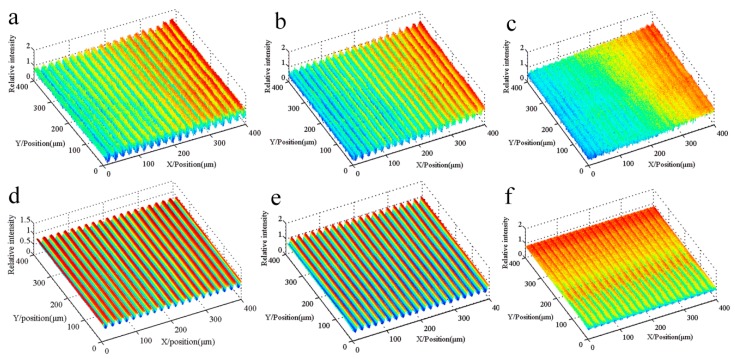
3D relative light intensity comparison between the common lithography and multi-repeated lithography with different alignment accuracies. (**a**–**c**) are the light intensities of the different focus errors, which correspond to (**d**–**f**), respectively, for the multi-repeated lithography.

Figure 11 shows the 3D relative light intensity comparison between the common lithography and multi-repeated lithography with different alignment accuracies. The area of the light intensity region is 400 µm × 400 µm. The data acquisition is completed though a CCD sensor, and the gray value of the image is normalized to form the relative intensity. Three different focus alignment positions are selected by the Sum-Modulus-Difference method(SMD) [14], which is the image definition evaluation algorithm. The intensity image is inclined in the x direction.

**Figure 12:**
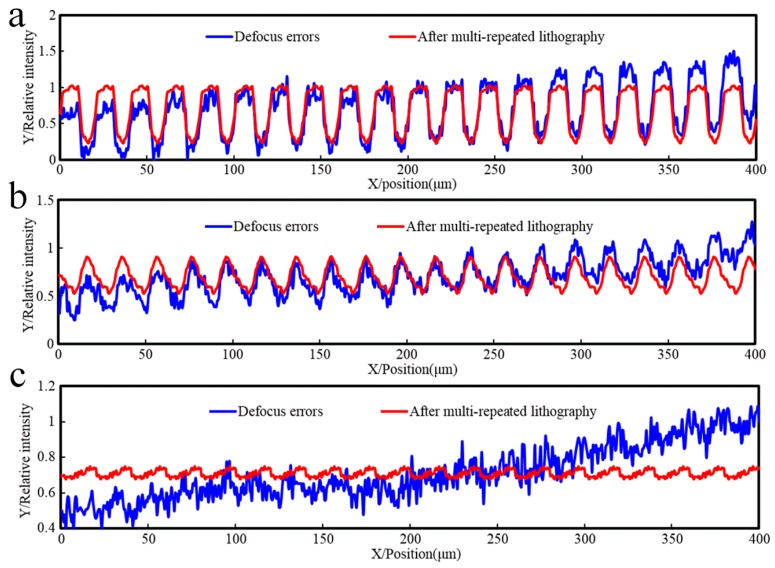
2D relative light intensity comparison between the common lithography and multi-repeated lithography with different alignment accuracies. The blue line represents the light intensity of the focal plane alignment error, and the red line represents the light intensity of multi-repeated lithography. (**a**) SMD = 1.00; (**b**) SMD = 0.51; (**c**) SMD = 0.01.

**Figure 13:**
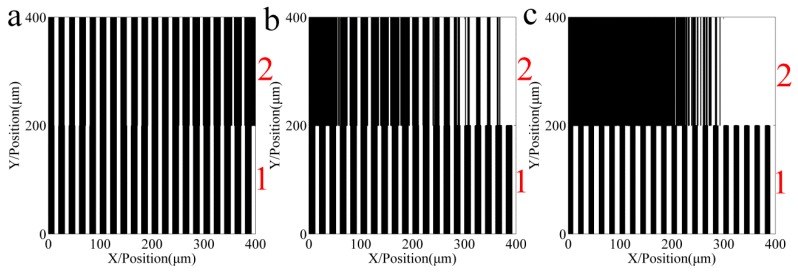
Developing results corresponding to the different alignment accuracies. Zones 1 and 2 are formed from the intensity of the multi-repeated lithography and the defocus errors, respectively. (**a**) Grating corresponding to “SMD = 1.00”; (**b**) Grating corresponding to “SMD = 0.51”; (**c**) Grating corresponding to “SMD = 0.01”.

Figure 12 shows the 2D relative light intensity comparison between the common lithography and multi-repeated lithography with different alignment accuracies. With an increase in the focal alignment error, the amplitude of the light intensity decreases; meanwhile, the curvature of the rising or falling curve decreases simultaneously. Assuming the parameter of threshold intensity is 0.56, the linear grating can be formed as in Figure 13, which shows that the developing results correspond with the spacing distance error.

It can be seen from these simulations that the multi-repeated method can generate a uniform light intensity distribution. With a decrease in the alignment precision, the maximum peak value of light intensity will be reduced, and the uniformity of the light intensity is not changed, which greatly increases the requirements for the sensitivity and threshold accuracy of the photoresist.

## 5. Experiment Results and Discussion

### 5.1. Experimental Setup

**Figure 14:**
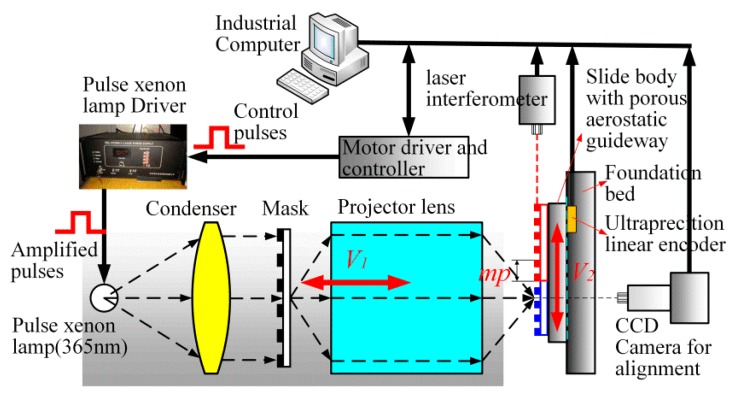
Schematic diagram for the experimental setup.

Figure 14 illustrates the grating lithography machine fabricated by Beijing Micro–Nano Precision Mechanical Co., Ltd. (BJJM, Beijing, China), in collaboration with Xi’an Jiaotong University. It can achieve a precision grating of 1250 mm. The whole assembly is placed on an isolated precision foundation. The device works in a room with temperature stabilization environment.

The grating lithography machine consists of four major elements: a reduced projection exposure system, a programmable illumination system, an AC servo rotary drive system with porous aerostatic guideways, and a fixed grating vacuum system.

Figure 14 shows that the lithography machine body with a natural granite bed in the $V_{2}$ direction of motion has porous aerostatic guide rails installed to ensure smooth and precision movement. An industrial CCD camera and a laser interferometer are used to align the multi-repeated projection lithography process.

**Figure 15:**
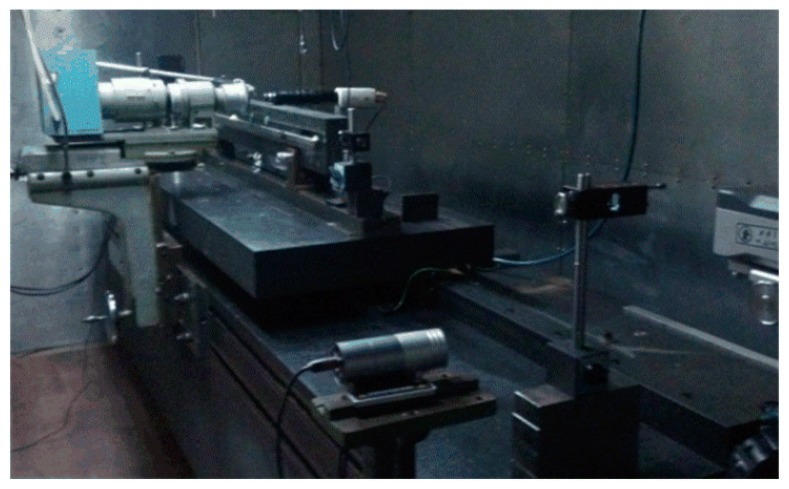
Photo of the experimental device.

To reduce the error caused by the loss of the experimental devices (See Figure 15), several principles for ultra-precision measurement and processing technology have been adopted:

(1) Abbe alignment criteria.
(2) Ultra-precision porous aerostatic bearing technology.
(3) Precision temperature sensor compensation.
(4) Pulse number error compensation.

**Figure 16:**
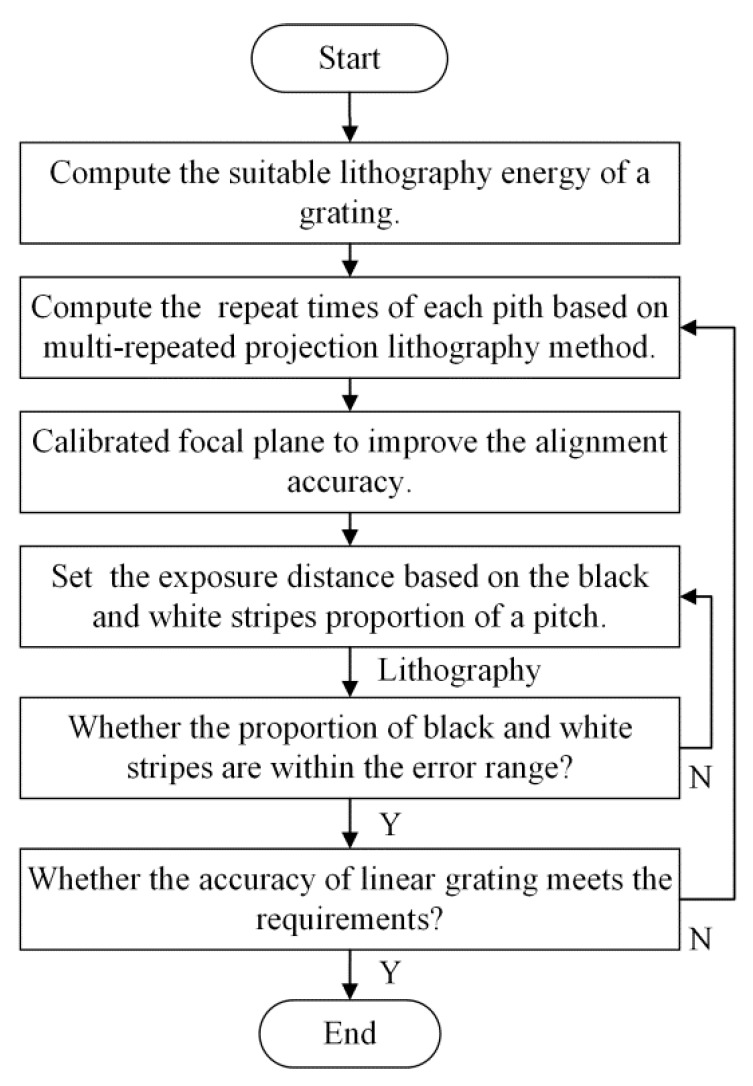
Flowchart of linear scale lithography based on multi-repeated process.

Figure 16 shows the flowchart of linear scale lithography based on the multi-repeated projection lithography process, in which the errors should be determined, and error compensation occurs to realize the high accuracy of the linear scale by changing the exposure distance with different repeat times, adjusting the workbench’s movement speed, and strengthening the uniformity of velocity in several cycles.

### 5.2. Pitch Accuracy

**Figure 17:**
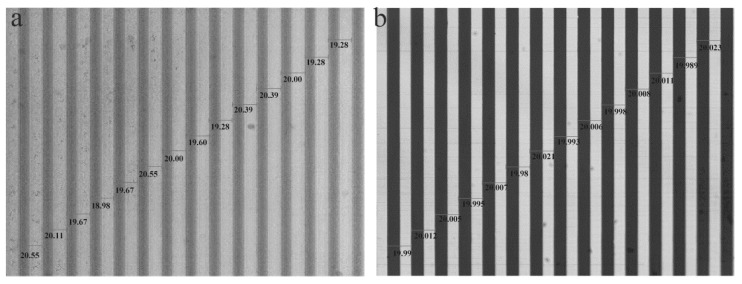
Image of pitch accuracy measurement. (**a**) A CCD image of pitch accuracy on the focal plane projected from the mask with errors by the projection lithography lens; (**b**) An image of pitch accuracy scanned from the linear scale, which is fabricated by the multi-repeated projection lithography based on the mask of (**a**).

**Figure 18:**
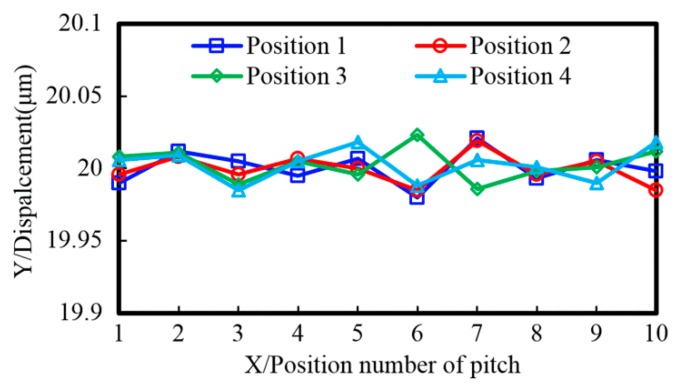
The pitch accuracy measurement of 10 arbitrary positions within 1 m long, and the pitch is 20 µm.

This section will provide some experimental results to verify the proposed projection lithography. The size of the linear scale is 1100 mm × 12 mm × 2 mm, and the nominal pitch is 20 µm. Errors including the pitch error on the mask and alignment error were introduced into the experiment, and then the repeated exposure number and spacing distance of non-integral pitch errors were considered to reduce and compensate the lithography errors. Figure 17 shows two images of pitch accuracy measurements. Figure 17a shows a CCD image of pitch accuracy on the focal plane that is projected from a mask with errors by the projection lithography lens; the maximum error is 20.55 µm, and the minimum error is 19.28 µm. Figure 17b shows an image of pitch accuracy that is scanned from the linear scale, which is fabricated by the multi-repeated projection lithography based on the mask with errors, and the minimum error is 41 nm. Figure 18 shows the measurement results of pitch accuracy, which are chosen from 10 consecutive adjacent pitches of four arbitrary positions in the linear scale. The maximum pitch error is small than 43 nm, and the average of four positions is between 20.00 µm and 20.03 µm.

### 5.3. Accuracy of a Linear Scale

**Figure 19:**
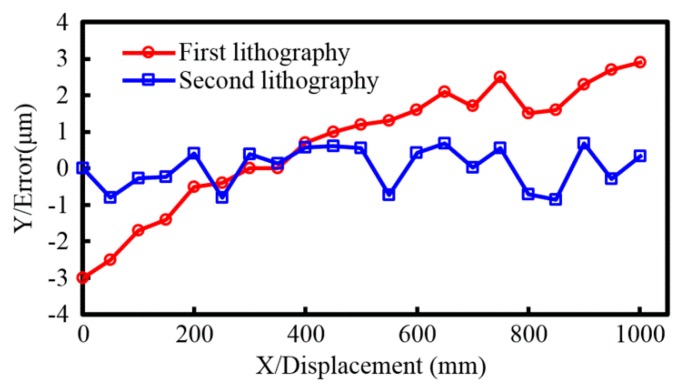
Accuracy comparison of the two multi-repeated projection lithography, the error of a linear scale that is fabricated from the first lithography is corrected and compensated to fabricate another linear scale using a second lithography.

The size of the linear scale manufactured by the multi-repeated projection lithography method is 1100 mm × 12 mm × 2 mm, the basis material is float glass, and the coefficient of linear expansion is 7.68 × 10^−6^/°C. Figure 19 shows the accuracy comparison of the two multi-repeated projection lithography; the error of a linear scale that is fabricated from the first lithography is corrected and compensated to fabricate another linear scale using a second lithography. During the experiment, the errors corresponding to the accurate position, the temperature and humidity variation curves, the ground vibration, and the electrical interference were programmed into the control system to improve the lithography accuracy. The data acquisition system for measurement accuracy includes a Heidenhain ND 287 display, MicroE1900 series reading head, and dual-frequency laser interferometer, and the measurement uncertainty is determined to be U = 0.3 µm. The room environment temperature is 20 ± 0.5 °C, and the humidity is less than 75% RH.

## 6. Conclusions

This study presented a multi-repeated projection lithography method for manufacturing an incremental linear scale. The common linear scale projection lithography splice process is replaced by the average homogenization technique, which overlaps the different position pitches regularly. The simulation and experimental results were discussed, and the following conclusions were drawn:

(1) The multi-repeat of the different grating positions with pitch error achieved a uniform light intensity distribution, which realized the homogenized error compensation of the whole length grating, making it so the lithography system reduces the requirements of mask accuracy.
(2) Increasing the exposure repetitions of the grating strips will reduce pitch errors, and when the exposure repetitions are equal to the number of pitches on the mask, the average homogenization error will be the minimum theoretically.
(3) The positioning error of the projection lithography motion system is considered to be one of the main factors affecting the exposed distance interval error, while the developing threshold of the exposure dose is constant, which is inversely proportional to the ratio of black stripes and white stripes, and the pitch is only slightly changed. It is necessary to adjust the developing threshold of exposure dose to make sure the ratio of black stripes and white stripes can reach the set value, which will directly affect the accuracy of the half cycle signal. On the other hand, the lithography control system needs the exposed distance interval error to be written in the control program, thus changing the ratio of black stripes and white stripes and achieve error compensation.
(4) The simulation analysis results confirmed that the light intensity with alignment error became more uniformly distributed using the multi-repeated lithography. Increasing the alignment error would reduce the maximum peak value of light intensity after the proposed method; the uniformity of the light intensity still was only slightly changed, which greatly increased the requirements for the sensitivity and the developing threshold accuracy of the photoresist.

According to the experimental results, when the pitch was set to be 20 µm, the multi-repeated process technique realized a pitch accuracy of 43 nm in any 10 locations of 1 m, and the accuracy of the whole grating was less than 1 µm/m. Meanwhile, when the experimental conditions follow higher requirements—*i.e.*, first, choosing a fine projection lithography lens adapted to a shorter wavelength, and second, decreasing the impact of environmental conditions, such as improving temperature accuracy, reducing ground vibration, and other constraints—it can be applied to manufacture a nanoscale-precision linear scale, and not just limited to the results presented herein.

## Figures and Tables

**Table 1:** Simulation results unit (µm).

**Spacing Distance**	19.7	19.8	19.9	20	20.1	20.2	20.3
**Pitch**	20	20	20	20	20	20	20
**Black grating**	9.69	9.87	9.97	10	9.97	9.87	9.69
**White grating**	10.31	10.13	10.03	10	10.03	10.13	10.31

## References

[1] Gerasimov F.M. (1967). Use of diffraction gratings for controlling a ruling engine. Appl. Opt..

[2] Flamand A.J., Bonnemason F., Thevenon A., Lerner J.M. (1989). The blazing of holographic gratings using ion-etching. Proc. SPIE.

[3] Yu H., Li X., Zhu J., Yu H., Qi X., Feng S. (2014). Reducing the line curvature error of mechanically ruled gratings by interferometric control. Appl. Phys. B.

[4] Liu H., Shi Y., Yin L., Jiang W., Lu B. (2013). Roll-to-roll imprint for high precision grating manufacturing. Eng. Sci..

[5] Lin B.J. (2006). Optical lithography—Present and future challenges. Competes Rendus Phys..

[6] Brunner T.A. (1988). Pattern-dependent overlay error in optical step and repeat projection lithography. Microelectron. Eng..

[7] Larramendy F., Blatche M.C., Mazenq L., Laborde A., Temple-Boyer P., Paul O. (2015). Microchannel-connected SU-8 honeycombs by single-step projection photolithography for positioning cells on silicon oxide nanopillar arrays. J. Micromech. Microeng..

[8] Bischoff J., Henke W., Werf J.V.D., Dirksen P. (1994). Simulations on step-and-scan optical lithography. Proc. SPIE.

[9] Williamson D.M., Mcclay J.A., Andresen K.W., Gallatin G.M., Himel M.D., Ivaldi J., Mason C., McCullough A., Otis C., Shamaly J. (1996). Micrascan III: 0.25-μm resolution step-and-scan system. Proc. SPIE.

[10] Ma X., Arce G.R. (2010). Techniques in computational lithography. Computational Lithography.

[11] Jain K., Zemel M., Klosner M. (2002). Large-Area High-Resolution Lithography and Photoablation Systems for Microelectronics and Optoelectronics Fabrication Jain. IEEE Proc..

[12] Lawes R.A. (2005). Manufacturing tolerances for UV LIGA using SU-8 resist. J. Micromech. Microeng..

[13] Mack C. (2007). Imaging Example: Dense Array of Lines and Spaces. Fundamental Principles of Optical Lithography: The Science of Microfabrication.

[14] Chern N.N.K., Neow P.A., Ang M.H. (2001). Practical issues in pixel-based autofocusing for machine vision. IEEE ICRA.

